# Calcified Aortic Aneurysm

**Published:** 1913-02

**Authors:** 


CALCIFIED AORTIC ANEURYSM.—Secousse, *Jour. de Med. de Bordeaux,* September 8, 1912. The patient died of acute broncho-pneumonia, the aneurysm being discovered post portem. It was 8 c. m. broad, 10 high, with walls about ½ c. m. thick. At each side was a diverticulum, of the size of a large nut, obstructed by a calculus.

